# Sexuality and gender identity in neurodevelopmental disorders

**DOI:** 10.1192/j.eurpsy.2026.10288

**Published:** 2026-07-31

**Authors:** K. M. Wilczyński

**Affiliations:** Department of Child and Adolescent Psychiatry, Medical University of Silesia, Sosnowiec, Poland

## Abstract

**Abstract:**

Autistic individuals face unique challenges across multiple domains of sexual and gender health that are often overlooked in clinical practice and education. This presentation will address four key areas: the significant gaps in sex education for autistic people, relationship difficulties and social-communication barriers that impact intimate partnerships, patterns of sexual orientation diversity in autism spectrum conditions, and the intersection of autism with transgender and gender-diverse identities.

Research indicates that autistic people receive inadequate or inaccessible sex education, often delivered in formats that don’t account for different learning styles or communication needs. Relationship challenges are common, stemming from difficulties with social reciprocity, sensory sensitivities, and navigating unwritten social rules in dating and intimacy. Studies show higher rates of diverse sexual orientations as well as gender dysphoria in autistic populations and due to social barriers and limited access to appropriate medical care and sexual education, this translates into higher rates of mental disorders and an increased risk of suicide.

**Disclosure of Interest:**

None Declared

